# Early-Onset Colorectal Adenocarcinoma and Multiple Metastases in an 11-Year-Old Patient With KRAS Mutation: A Case Report

**DOI:** 10.7759/cureus.81235

**Published:** 2025-03-26

**Authors:** Mohammad Rezazadeh, Ahmadreza Kheradpishe, Amirreza Kamyabi, Bahareh Shateri Amiri, Mohammad Faranoush

**Affiliations:** 1 Student Research Committee, Faculty of Medicine, Iran University of Medical Sciences (IUMS), Tehran, IRN; 2 Internal Medicine, School of Medicine, Hazrat-e Rasool General Hospital, Iran University of Medical Sciences (IUMS), Tehran, IRN; 3 Pediatric Oncology, Pediatric Growth and Development Research Center, Institute of Endocrinology, Iran University of Medical Sciences (IUMS), Tehran, IRN

**Keywords:** case report, early-onset colorectal cancer, kras mutation, malignancy, multiple organ metastasis

## Abstract

Early-onset colorectal cancer (EO-CRC), defined as colorectal adenocarcinoma diagnosed before age 50, is increasing globally, with distinct clinicopathologic and molecular features compared to adult-onset disease. While risk factors such as genetic mutations and lifestyle influences are implicated, pediatric cases remain exceptionally rare. This report presents a unique case of colorectal adenocarcinoma with multiple metastases in an 11-year-old patient, contributing critical insights into the molecular pathogenesis and diagnostic challenges of EO-CRC in pediatric populations. Furthermore, the aggressive nature of this disease underscores significant treatment challenges, ranging from limited evidence-based therapeutic protocols for young patients to variable responses to multimodal therapies, resulting in a generally poor prognosis.

## Introduction

Colorectal cancer (CRC) is a significant global health burden, ranking as the third most prevalent cancer and cause of cancer-related mortality in both men and women. In 2020, CRC claimed the lives of 576,858 individuals worldwide [1]. Early-onset colorectal cancer (EO-CRC) is defined as the development of CRC before the age of 50. CRC predominantly affects older populations. However, EO-CRC incidence has seen a concerning rise over the past few decades [2]. The most prevalent anatomical sites for EO-CRC are the rectum (35%-37%), distal colon (25%-26%), and proximal colon (22%-23%) [3].

The etiology of EO-CRC is multifactorial, with various potential risk factors implicated in its pathogenesis. While it is hypothesized that certain lifestyle factors, such as an unhealthy dietary pattern, alcohol consumption, and tobacco use, may contribute to the development of EO-CRC by inducing colonic inflammation and disrupting the gut microbiome, studies suggest that 14%-25% of patients with early-onset disease have a pathogenic germline variant associated with cancer risk [4]. Furthermore, young symptomatic individuals often delay seeking medical attention for up to six months, potentially allowing the disease to progress unchecked [5]. Poorly differentiated histology and aggressive subtypes like mucinous and signet ring cell carcinomas are more commonly encountered in EO-CRC cases.

Carcinogenesis in colorectal carcinomas is primarily caused by the activation of the RAS-RAF-MEK-ERK signaling cascade, which promotes cell migration, differentiation, and proliferation in various tumors. The KRAS, NRAS, and BRAF gene mutations are the most common manifestations [6]. One-third of all malignancies include RAS mutations, with KRAS mutations being the most common (more than 80%), while NRAS mutations are very uncommon (about 10%) [7]. KRAS codon 12/13 mutations are common in various cancers, especially in CRC. G12D and G12V are frequent KRAS mutations associated with pancreatic cancer and CRC. Interestingly, some KRAS variants (around 20%) carry a reactive cysteine at the 12th amino acid position. KRAS mutation status is used in the clinical setting to inform systemic treatment options and predict oncologic outcomes [8].

This report presents a rare case of an 11-year-old patient diagnosed with EO-CRC and multiple metastases. Through the description of this case, we aim to provide important new information to the corpus of research about the unusually early beginning of the disease, its rapid development, and the clinical difficulties in managing EO-CRC in pediatric patients.

## Case presentation

**Figure 1:**
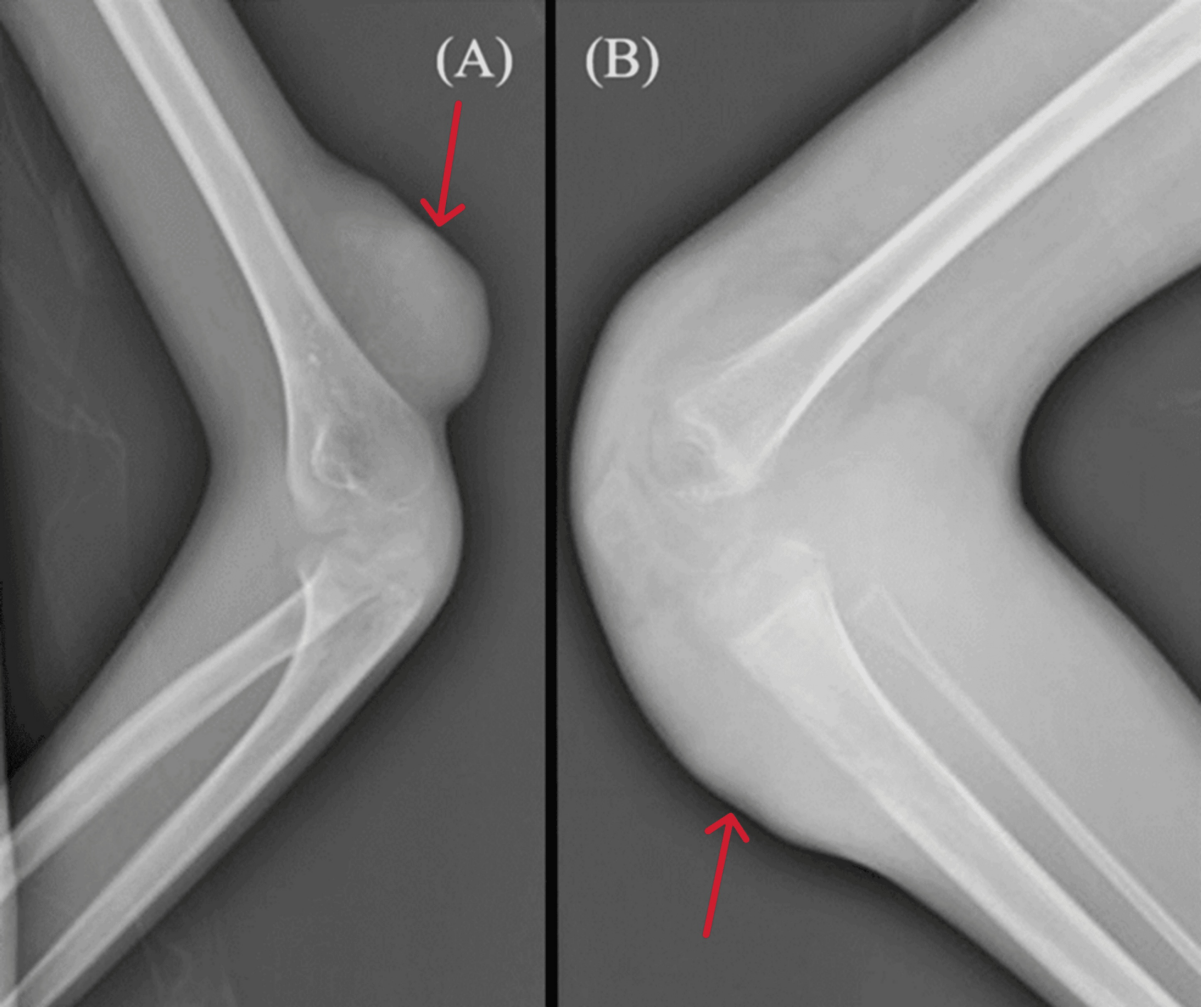
X-ray showing a large mass of soft tissue with vascularity and subcutaneous soft tissue edema and increased density of subcutaneous fat in the left elbow (A) and right knee (B) suggestive of hemangioma (red arrows)

An 11-year-old boy with a history of multiple soft tissue hemangiomas since infancy, chronic constipation for more than three months, and no surgical and drug history presented to the emergency department with symptoms including intermittent rectorrhagia, passing fresh blood, progressive hypogastric pain, headache, nausea, vomiting, and loss of appetite in the last month. He had no family history of gastrointestinal malignancies. Physical examination revealed cutaneous hemangiomas on the left elbow and right knee (Figure 1), along with lesions suggestive of pilomatricoma on his left heel. His sclerae appeared icteric, and he presented with malnourishment (height: 145 cm; Z-score: 0.2; weight: 27 kg; Z-score: -1.7). Physical examination did not yield any other relevant alteration except decreased bowel sounds and severe tenderness on the left lower quadrant in the abdominal examination. The patient was conscious and stable hemodynamically, with a normal digital rectal examination. Vital signs upon admission were within normal limits (blood pressure 100/60 mmHg, pulse rate 80 beats per minute, respiratory rate 16 breaths per minute, temperature 36.8°C, and oxygen saturation 98%). Blood tests at presentation showed an increase in tumor markers (carcinoembryonic antigen: 9.6 ng/L; carbohydrate antigen 19-9: 412.3 U/mL), total bilirubin (2.7 mg/dL), direct bilirubin (0.8 mg/dL), liver enzymes (aspartate transferase, AST: 143 IU/L; alanine transferase, ALT: 220; alkaline phosphatase, ALP: 151 IU/L), and C-reactive protein (23 mg/L). Pancytopenia (white blood cells: 0.5 × 1,000/mm^3^; hemoglobin: 8.3 g/dL; platelets: 9 × 1,000/mm^3^) was also evident in the results of blood tests (Table 1).

**Table 1:** Laboratory results at the presentation and the last hospitalization WBC: white blood cell; RBC: red blood cell; Hb: hemoglobin; BUN: blood urea nitrogen; AST: aspartate transferase; ALT: alanine transferase; ALP: alkaline phosphatase; CA19-9: carbohydrate antigen 19-9; CEA: carcinoembryonic antigen

Parameter	At presentation	Last hospitalization	Units	Reference value
WBC	0.5	5.74	×1,000/mm^3^	4-10
RBC	2.87	3.16	mill/mm^3^	4.5-6.3
Hb	8.3	9.6	g/dL	12-16
Platelet	9	7	×1,000/mm^3^	140-440
BUN	18	20	mg/dL	5-23
Creatinine	1.3	0.5	mg/dL	0.5-1.5
AST	143	154	IU/L	5-40
ALT	220	203	IU/L	5-40
ALP	151	1,936	IU/L	180-1,200
Total bilirubin	2.7	9.7	mg/dL	0.1-1.2
Direct bilirubin	0.8	7.4	mg/dL	0-0.4
CA19-9	412.3	-	U/mL	0-37
CEA	9.6	-	ng/L	0-2.5
Sodium	118	142	mEq/L	136-145
Potassium	2.6	2.9	mEq/L	3.7-5.5
Calcium	6.7	8.3	mEq/L	8.6-10.6
C-reactive protein	23	31	mg/L	8-10

**Figure 2:**
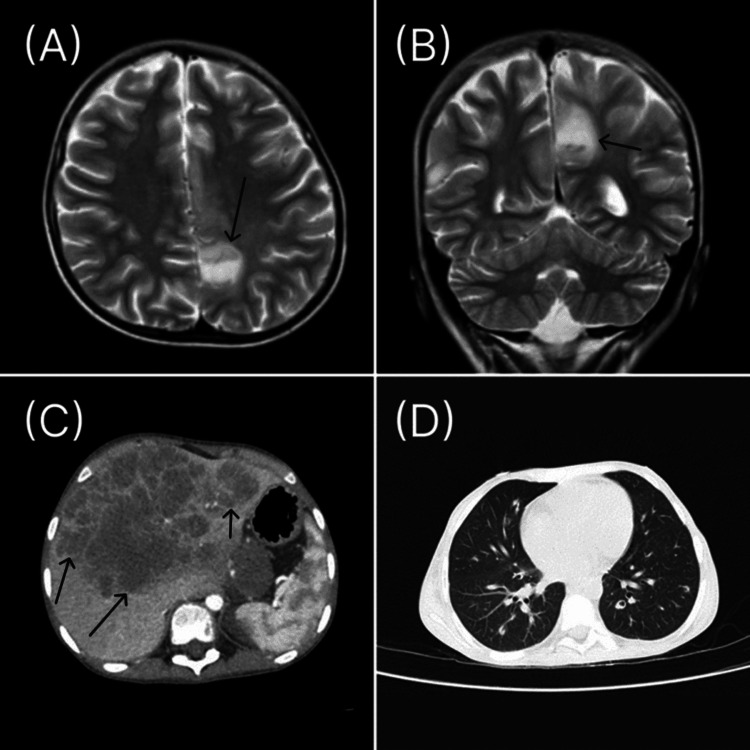
(A,B) MRI T2-weighted image of the brain. The metastatic lesion showed a high signal in the left subcortical area of horizontal and coronal sections. (C) CT scan of the abdomen with contrast shows a large lobulated mass in the liver suggestive of metastasis with invasion to the inferior vena cave. (D) Mediastinal and lung windows of axial CT scan of the chest show numerous nodules with speculated margins and multiple cavities in the chest MRI: magnetic resonance imaging; CT: computed tomography

An abdominopelvic MRI with contrast showed enhancing masses with extension to and involvement of the mesorectal fat at the rectosigmoid junction, which suggested colorectal adenocarcinoma. Colonoscopy showed two polyps at the rectosigmoid junction, measuring 3 x 3 x 4 cm and 3 x 3 x 2 cm. The biopsy showed a high-grade dysplastic neoplasm, and the histopathological diagnosis was tubulovillous adenocarcinoma (pT4bpN2bpM1). The molecular assay amplified KRAS codon 13 mutations (c.38G>A; p.Gly13Asp), and the polymerase chain reaction identified wild-type NRAS and BRAF. The patient was scheduled for surgical resection of the polyps. However, before the operation, he complained of severe abdominal pain, constipation, and inability to pass gas. Surgical intervention was performed due to intestinal volvulus. A postoperative computed tomography (CT) scan revealed multiple necrotic masses with peripheral enhancement in the body and tail of the pancreas, which were suggestive of metastasis. Metastases were also identified in the left adrenal gland and liver (Figure 2).

During hospitalization, the patient experienced episodic hallucinations and transient aphasia. Brain MRI revealed one metastatic lesion in the left subcortical area. Chest CT scan showed cavitary nodules in the lungs (Figure 2). X-rays of lower limb bones demonstrated evidence of metastatic disease. Following the results of imaging and pancytopenia, a bone marrow biopsy was performed that showed diffuse bone marrow replacement by metastatic adenocarcinoma.

Due to the advanced stage of the disease, the patient underwent palliative chemotherapy (oxaliplatin, folinic acid, leucovorin, and 5-fluorouracil) for three months. The patient developed symptoms, including fever and bloody diarrhea. Blood tests indicated a decrease in hemoglobin (9.6 g/dL) and platelet count (7 × 1,000/mm^3^), as well as an increase in direct bilirubin (7.4 mg/dL), total bilirubin (9.7 mg/dL), liver enzymes (AST: 154 international units, IU/L; ALT: 203 IU/L; ALP: 1,936 IU/L), and C-reactive protein (31 mg/L), as shown in the results of blood tests (Table 1). The patient ultimately succumbed to death due to cardiorespiratory arrest.

## Discussion

This case report presents a rare and aggressive case of CRC metastasizing to multiple organs, including the pancreas, brain, liver, lungs, and bones, in an 11-year-old Iranian boy with a history of multiple skin hemangiomas. EO-CRC has distinct pathophysiologic mechanisms compared to adult-onset CRC, as it is largely independent of lifestyle factors. Key risk factors include a family history of CRC and inflammatory bowel disease, but lifestyle and environmental influences like obesity, unhealthy diets, processed meats, and sugar-sweetened beverages also contribute to the pathogenesis of EO-CRC [9]. Patients with EO-CRC generally have a poor prognosis, with reported five-year survival rates less than 25%. Factors such as disease extent, distant metastases, and overall health status can significantly impact survival outcomes [10]. A case series of seven pediatric CRC patients found frequent symptoms, including abdominal pain, vomiting, intestinal obstruction, and rectal bleeding [11]. A case of a 40-year-old patient with KRAS amplification was reported, showing primary resistance to monoclonal antibody therapy (cetuximab) combined with chemotherapy. This highlights the importance of genetic testing in treatment planning by indicating that higher KRAS gene copy numbers may be linked to treatment resistance and poor outcomes in CRC [12]. Another report concluded that KRAS gene copy number variations can serve as a negative predictive biomarker for the efficacy of cetuximab in metastatic EO-CRC. The patient demonstrated increased KRAS gene copy numbers during treatment, correlating with resistance to treatment [13]. O'Reilly et al. conducted a genomic and transcriptomic analysis of a patient with EO-CRC, identifying a KRAS G12D mutation, TP53 mutation, and other significant chromosomal deletions. These findings emphasize the need for personalized molecular profiling in EO-CRC, particularly in cases with rare genomic rearrangements, to guide treatment decisions and surveillance. They showed how certain mutations, such as those involving the WRN gene, can influence responses to chemotherapeutic agents like 5-fluorouracil [14].

Mutations in the KRAS gene, as seen in this case, are present in approximately 40% of CRCs and have been associated with more aggressive disease behavior, increased metastatic potential, and poorer prognosis. Patients with CRC with tumors harboring a KRAS gene mutation often do not respond to conventional therapies [15]. Additionally, several studies suggest a potentially higher prevalence of KRAS mutations in EO-CRC patients [16,17].

The pattern of metastatic spread is crucial for treatment decisions. Common sites of CRC metastasis include the peritoneum (70%), thorax (21%), and liver (10%) [18]. Brain metastasis is rare, occurring in 2.10% of CRC patients. Patients with brain metastases have a poor prognosis and can develop severe neurological complications, such as seizures, cognitive impairment, and focal neurological deficits [19]. Management of CRC in pediatric patients often involves a multimodal approach, including chemotherapy (70.1%), surgery (54.1%), and radiation therapy (19.5%) [20]. However, advanced or metastatic disease treatment remains a significant challenge, especially in cases with widespread dissemination to multiple organs.

Given the growing emphasis on precision oncology, broader next-generation sequencing (NGS) to identify targetable mutations and germline testing for hereditary syndromes would have been invaluable in this case. While our analysis confirmed a KRAS mutation, NGS could have clarified eligibility for immunotherapy or emerging targeted therapies, and germline testing might have uncovered a predisposing syndrome, enabling genetic counseling and family screening despite the absence of reported familial malignancies.

## Conclusions

This case documents an exceptionally rare pediatric EO-CRC with aggressive features and KRAS mutation. The rapid progression and treatment resistance highlight diagnostic challenges and the need for improved strategies. Pediatric patients with gastrointestinal symptoms should have CRC considered in differential diagnosis. Comprehensive molecular profiling is essential for guiding treatment decisions. Future research should focus on understanding molecular mechanisms and developing better treatment options.
